# Extremity fractures, attempted suicide, blood transfusion and thromboembolic events are independent risk factors for a prolonged hospital stay in severely injured elderly

**DOI:** 10.1007/s40520-024-02817-4

**Published:** 2024-08-07

**Authors:** Philipp Störmann, Jason A. Hörauf, Ramona Sturm, Lara Zankena, Jonin Serafin Zumsteg, Rolf Lefering, Ingo Marzi, Hans-Christoph Pape, Kai Oliver Jensen, TraumaRegister DGU

**Affiliations:** 1 Department of Trauma, Hand and Reconstructive Surgery, Goethe University Frankfurt, University Hospital, Frankfurt, Germany; 2 Department of Trauma, University Hospital Zurich, Rämistrasse 100, CH, 8091 Zurich, Switzerland; 3 Institute for Research in Operative Medicine (IFOM), University Witten/Herdecke, Cologne, Germany

**Keywords:** Polytrauma, Geriatrics, Length of stay, Severely injured, Prolonged LOS

## Abstract

**Methods:**

Due to demographic change, the number of polytraumatized geriatric patients (> 64 years) is expected to further increase in the coming years. In addition to the particularities of the accident and the associated injury patterns, prolonged inpatient stays are regularly observed in this group. The aim of the evaluation is to identify further factors that cause prolonged inpatient stays.

A study of the data from the TraumaRegister DGU® from 2016–2020 was performed. Inclusion criteria were an age of over 64 years, intensive care treatment in the GAS-region, and an Injury Severity Score (ISS) of at least 16 points. All patients who were above the 80th percentile for the average length of stay or average intensive care stay of the study population were defined as so-called long-stay patients. This resulted in a prolonged inpatient stay of > 25 days and an intensive care stay of > 13 days. Among other, the influence of the cause of the accident, injury patterns according to body regions, the occurrence of complications, and the influence of numerous clinical parameters were examined.

**Results:**

A total of 23,026 patients with a mean age of 76.6 years and a mean ISS of 24 points were included. Mean ICU length of stay was 11 ± 12.9 days (regular length of stay: 3.9 ± 3.1d vs. prolonged length of stay: 12.8 ± 5.7d) and mean inpatient stay was 22.5 ± 18.9 days (regular length of stay: 20.7 ± 15d vs. 35.7 ± 22.3d). A total of n = 6,447 patients met the criteria for a prolonged length of stay. Among these, patients had one more diagnosis on average (4.6 vs. 5.8 diagnoses) and had a higher ISS (21.8 ± 6 pts. vs. 26.9 ± 9.5 pts.)

Independent risk factors for prolonged length of stay were intubation duration greater than 6 days (30-fold increased risk), occurrence of sepsis (4x), attempted suicide (3x), presence of extremity injury (2.3x), occurrence of a thromboembolic event (2.7x), and administration of red blood cell concentrates in the resuscitation room (1.9x).

**Conclusions:**

The present analysis identified numerous independent risk factors for significantly prolonged hospitalization of the geriatric polytraumatized patient, which should be given increased attention during treatment. In particular, the need for a smooth transition to psychiatric follow-up treatment or patient-adapted rehabilitative care for geriatric patients with prolonged immobility after extremity injuries is emphasized by these results.

## Introduction

The proportion of Germanys population older than 65 years of age is expected to rise to 34% by the year 2034 and similar developments are reported for other developed countries [1, 2]. In addition, older patient’s lifestyle changed to higher activity levels, which also results in a higher number of accidents and more elderly trauma patients [3, 4]. In this context, it has been demonstrated in recent years that the principles of care for younger patients cannot be readily applied in the treatment of severely injured elderly [5]. In addition to differences in accident mechanisms and the influence of co-morbidities as well as long-term medication, physiological changes, such as the response to blood loss, are also difficult to compare with a younger collective. For these reasons, changes have already been made in the treatment of this subgroup. For example, it is recommended that higher age alone should be considered as a criterion for trauma team activation and that special teams should be established for the treatment of geriatric trauma [3, 6]. It is well-known that increasing age is an independent risk factor for mortality regardless of injury severity, where there is an increased risk of mortality, especially if the patient is older than 74 years of age [7–9]. Furthermore, the Revised Injury Severity Classification (RISC) II score demonstrated that age greater than 55 years makes an independent contribution to outcome that increases with age [10].

Length of hospital stay, along with mortality, is used as a quality criterion for the care of severely injured patients. Here, the Injury Severity Score (ISS), the injury pattern, the occurrence of complications, and the presence of pre-existing conditions is assumed to increase resource use and length of stay on Intensive Care Units (ICU) as well as the overall in-patient stay [11, 12]. Due to the numerous concomitant circumstances complicating treatment in the elderly, one might assume that especially severely injured geriatric patients are more likely to undergo a complicated long-term stay in intensive care and prolonged overall hospitalization [13]. Especially in the complex treatment of severely injured elderly patients, consequently longer courses seem likely to occur and cause excessive resource consumption and high costs.

Therefore, in this study, we focused on the identification of independent risk factors that might prolong the ICU-LOS and overall duration of hospitalization in severely injured elderly.

### Methods

#### Data

The TraumaRegister DGU® (TR-DGU) of the German Trauma Society (Deutsche Gesellschaft für Unfallchirurgie, DGU) was founded in 1993. The aim of this multi-center database is a pseudonymized and standardized documentation of severely injured patients.

Data are collected prospectively in four consecutive time phases from the site of the accident until discharge from hospital: A) Pre-hospital phase, B) Emergency room and initial surgery, C) Intensive care unit and D) Discharge. The documentation includes detailed information on demographics, injury pattern, comorbidities, pre- and in-hospital management, course on intensive care unit, relevant laboratory findings including data on transfusion and outcome of each individual. The inclusion criterion is admission to hospital via emergency room with subsequent ICU/IMC care or reach the hospital with vital signs and die before admission to ICU. The infrastructure for documentation, data management, and data analysis is provided by AUC–Academy for Trauma Surgery (AUC—Akademie der Unfallchirurgie GmbH), a company affiliated to the German Trauma Society. The scientific leadership is provided by the Committee on Emergency Medicine, Intensive Care and Trauma Management (Sektion NIS) of the German Trauma Society. The participating hospitals submit their data pseudonymized into a central database via a web-based application. Scientific data analysis is approved according to a peer review procedure laid down in the publication guideline of TraumaRegister DGU®.

The participating hospitals are primarily located in Germany (90%), but a rising number of hospitals of other countries contribute data as well (at the moment from Austria, Belgium, China, Finland, Luxembourg, Slovenia, Switzerland, The Netherlands, and the United Arab Emirates). Currently, more than 28,000 cases from almost 700 hospitals are entered into the database per year. Participation in TR-DGU is voluntary. For hospitals associated with TraumaNetzwerk DGU®, however, the entry of at least a basic data set is obligatory for reasons of quality assurance. Given the background of quality assurance and anonymized data, informed consent is not necessary, which is confirmed by the review board of the official trauma registry of the german society of trauma surgery. Initial data and privacy agreements are obtained from the participating hospitals. The study was performed in accordance with the Declaration of Helsinki and following STROBE guidelines and the RECORD guidelines for observational studies (Reporting of studies Conducted using Observational Routinely Collected Data).

### Inclusion and exclusion criteria

This retrospective cohort study included patients from the TR-DGU who were treated in participating German speaking trauma centers (Countries: Germany, Austria, Switzerland; GAS-Regions) between 01/2016 and 12/2020. We included hospitals from the GAS-Regions due to their homogenous medical care quality. The study period was chosen due to the fact, that some parameters were introduced in the questionnaire of the database since 2016. We wanted to examine a cohort with geriatric, severely injured patients. Therefore, this study only included patients aged 65 years or older with an ISS of at least 16 points. We excluded patients who were early transferred out (< 48 h) or transferred in with a delay of more than 2 days due to incomplete patient records and patients without ICU treatment. Patients who died or were transferred within the regular stay were excluded since there were no data available afterwards and could not be evaluated with respect to a prolonged stay.

The present study is in line with the publication guidelines of the TraumaRegister DGU® and registered as TR-DGU project ID 2021–030.

We defined our threshold for a prolonged stay (intensive care unit and hospital) on the distribution of the length of stay of the overall collective of severely injured elderly. Patients with a length of stay (LOS) above the 80% percentile were defined as the long-term group. This resulted in more than 13 days for the ICU LOS, and more than 25 days for hospital LOS. For patients who did not meet the criteria for an extended stay, deceased and transferred patients were subsequently excluded.

Coagulopathy was considered present if at least one of the following conditions were found on admission: Quick’s value ≤ 60%, or PTT ≥ 40 s, or International Normalized Ratio (INR) ≥ 1.4.

### Statistical analysis

Continuous variables are presented with mean and standard deviation (SD), or as median with interquartile range (IQR, 1st and 3rd quartile) in case of a rather skewed distribution. Categorical variables are presented with number and percentage. Due to the large number of cases, formal statistical comparison of the two study groups was avoided since even minor differences (± 1%, or ± 0.05 SD) would turn out to be significant (p < 0.05).

Risk factors for a prolonged stay (hospital and/or ICU) were investigated with a multivariate logistic regression model. The independent predictors are listed in Table 5. Results are presented as odds ratios (OR) with a 95% confidence interval (CI).

Statistical analysis was performed using SPSS statistical software (version 25, IBM Inc., Armonk, NY, USA).

## Results

**Fig. 1:**
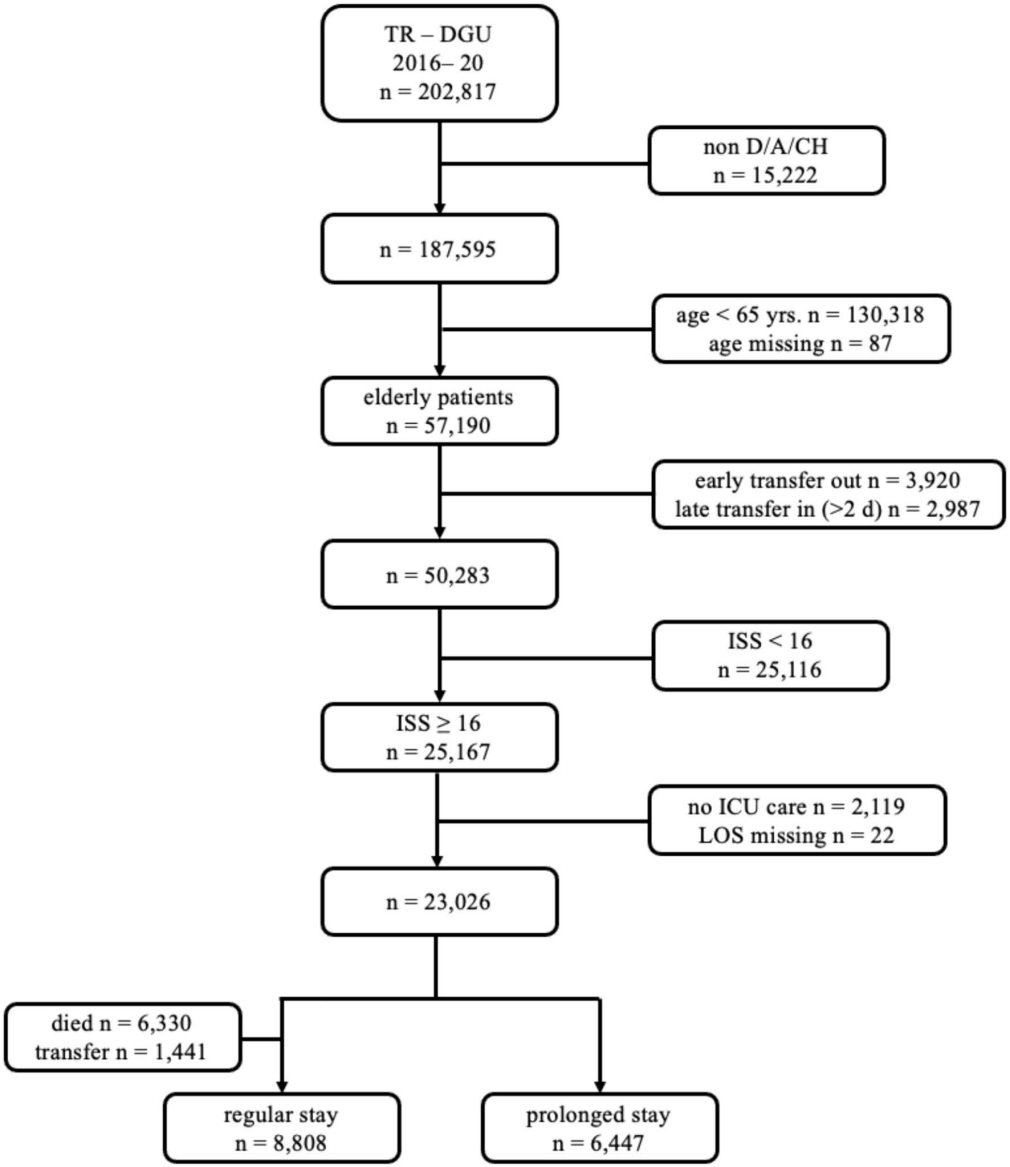
Definition of the study population (*D/A/CH* Germany/Austria/Switzerland, *yrs*. years, *ISS* injury severity score, ICU intensive care unit, *LOS* length of stay)

A total of 202,817 patients from the TR-DGU were checked for eligibility. The median hospital length of stay was 18 [11–28] days. A total of 6,335 deceased (median length of stay 2 [1–6] days) were excluded from further analysis just as 1,441 were transferred (LOS: 9 [6–13] days). After those exclusions, n = 15,255 geriatric patients aged 65 years or more qualified for analysis (Fig. 1). Of these patients, n = 8808 had a regular length of stay while n = 6447 (42.2%) met the definition for a prolonged stay (Tab. 1).

**Table 1:** Basic demographic patient data of patients with and without prolonged LOS

	Regular LOS *n* = 8808	Prolonged LOS *n* = 6447	Total
age (years), mean ± SD	76.9 ± 7.7	76.3 ± 6.9	76.6 ± 7.4
age ≥ 70 years (%)	78 (*n* = 6866)	79 (*n* = 5,095)	78.4 (*n* = 11,961)
male (%)	59.2 (*n* = 5216)	66.4 (*n* = 4282)	62.3 (*n* = 9.498)
blunt trauma (%)	98.8 (*n* = 8333)	98.2 (*n* = 6029)	98.5 (*n* = 14,362)
ASA 3 and 4 (%)	39.7 (*n* = 3260)	45.2 (*n* = 2649)	42 (*n* = 5909)
AIS_head_ ≥ 3 (%)	57.3 (*n* = 5051)	60 (*n* = 3870)	58.5 (*n* = 8921)
AIS_chest_ ≥ 3 (%)	49.4 (*n* = 4353)	54.9 (*n* = 3.538)	51.7 (*n* = 7891)
AIS_abdomen_ ≥ 3 (%)	7 (*n* = 619)	11 (*n* = 710)	8.7 (*n* = 1329)
AIS_extremities_ ≥ 3 (%)	16.7 (*n* = 1475)	29.9 (*n* = 1925)	22.3 (*n* = 3400)
isolated TBI (AIS_head_ ≥ 3) (%)	23.4 (*n* = 2064)	19.7 (*n* = 1271)	21.9 (*n* = 3335)
ISS (points), mean ± SD	21.8 ± 6.1	26.9 ± 9.6	24.0 ± 8.2
Polytrauma Berlin definition (%)	27.0 (n = 2378)	45.2 (n = 2917)	34.7 (*n* = 5295)
number of diagnoses, mean ± SD	4 [3–6]	6 [4–8]	5 [3–7]

*LOS* length of stay, *ASA *American society of anesthesiologists, *AIS *abbreviated injury scale, *TBI *traumatic brain injury, *ISS* Injury severity score, *SD* standard deviation)

Mean age was 76.6 years with 75% (n = 11,961) of the patients being older than 70 years of age, where age was comparable between groups (regular LOS 76.9 yrs. vs prolonged stay 76.3 yrs.). 62.3% (n = 9.498) of the patients were male, with a higher proportion of male patients in the group with prolonged stay (regular stay 59.2% vs. prolonged stay 66.4%). The mean Injury Severity Score (ISS) was 24.0 ± 8.2 points, and 98.5% (n = 14,362) suffered a blunt trauma. 34.7% of the patients had multiple injuries according to the Berlin Definition of polytrauma (regular stay: 27.0% vs. prolonged stay: 45.2%) with a mean of 5.16 trauma-specific diagnoses. Higher ISS and more patients fulfilling the Berlin Definition of polytrauma were recorded for the prolonged LOS group, additionally the rate of lower extremity fractures in these patients was almost twice as high. Basic patient data is presented in Table 1 (Tab. 1).

Most common mechanism of injury were falls from lower heights (44%), while 37% were involved in traffic accidents. Attempted suicide was registered in 1.9% of all patients and was more frequently seen in patients with prolonged LOS (prolonged LOS 3.5% vs. 0.8% regular LOS). Details regarding the mechanism of trauma are listed in Table 2.

**Table 2:** Mechanism of trauma. (LOS: length of stay)

	Regular LOS	Prolonged LOS	Total
Mechanism of injury			
Motor vehicle accident (%)	12.2 (*n* = 1062)	16.1 (n = 1026)	13.8 (*n* = 2088)
Motorcycle accident (%)	4.3 (*n* = 374)	4.9 (n = 312)	4.5 (n = 686)
Bicycle accident (%)	12.8 (*n* = 1,118)	10.7 (n = 680)	11.9 (*n* = 1798)
Pedestrian (%)	4.7 (*n* = 406)	9.9 (n = 630)	6.9 (*n* = 1036)
High fall (%)	13.3 (*n* = 1160)	15.2 (*n* = 972)	14.1 (n = 2,132)
Low fall (%)	48.2 (*n* = 4206)	37.8 (*n* = 2411)	43.8 (*n* = 6617)
Assault/violence (%)	0.7 (*n* = 29)	0.6 (*n* = 24)	0.7 (*n* = 53)
Attempted suicide (%)	0.8 (*n* = 66)	3.5 (*n* = 220)	1.9 (*n* = 286)

A higher rate of patients that presented with longer stays were unconscious on scene (Glasgow Coma Scale, GCS ≤ 8 points): 6.8% regular LOS vs. 23.8% prolonged LOS (Table 3). Also shock on scene (regular LOS 4.3% vs. 11.4%) and shock on admission (regular LOS 3.8% vs. 12.9%) were observed more frequently in the prolonged LOS group. About half of the patients were on permanent platelet inhibition (46.7%), 23.4% used DOAC and 20.4% were on Vitamin-K-antagonists. While non-survivors were excluded from the regular LOS group, hospital mortality was 15.2% (n = 977) in patients with a prolonged stay.

**Table 3:** Prehospital data

	Regular LOS	Prolonged LOS	Total
GCS 3–8 on scene (%)	6.8 (*n* = 523)	23.8 (*n* = 1327)	14 (*n* = 1850)
Pre-hospital shock (SBP ≤ 90 mmHg) (%)	4.3 (*n* = 313)	11.4 (*n* = 601)	7.2 (*n* = 914)
Shock at admission (SBP ≤ 90 mmHg) (%)	3.8 (*n* = 312)	12.9 (*n* = 776)	7.6 (*n* = 1088)
Coagulopathy (%)	47.2 (*n* = 3532)	49.7 (n = 2511)	48.2 (n = 6,043)
Positive blood alcohol (%)	15.2 (*n* = 277)	16.2 (*n* = 289)	15.7 (*n* = 566)
Pre-medication with Vitamin-K-antagonist (%)	18.3 (*n* = 645)	23.3 (*n* = 586)	20.4 (*n* = 1231)
Pre-medication with DOAC (%)	23 (*n* = 813)	24 (*n* = 603)	23.4 (*n* = 1416)
Pre-medication with PAI (%)	48 (*n* = 1696)	44.9 (*n* = 1127)	46.7 (*n* = 2823)

*LOS *length of stay, *GCS* glasgow coma scale, *SBP* systolic blood pressure, *DOAC* direct acting oral anticoagulants, *PAI* platelet aggregation inhibition)

Mean/median length of ICU stay was 10.0/6 days and mean/median length of hospital stay was 22.5/18 days. The necessity of mechanical ventilation on ICU was given in 43.8% of all patients, where the rate of mechanical ventilation (regular LOS: 20.7% vs. prolonged LOS 75.4%) was higher in the group with prolonged stay. If mechanical ventilation was required, duration of ventilation (regular LOS: 0.5 ± 1.7 d vs. prolonged mechanical ventilation 11.7 ± 13.3 days) higher for patients with longer stays. Multiple organ failure occurred in 44.5% of patients with a prolonged stay (vs. 6.6% with regular LOS) (Tab. 4).

**Table 4:** Outcome parameter

	Regular LOS	Prolonged LOS	Total
Mechanical ventilation (%)	20.7 (*n* = 1826)	75.4 (*n* = 4858)	43.8 (*n* = 6684)
Duration of ventilation (days), median [IQR]	1 [1–4]	14 [6–22]	9 [2–18]
Multiple organ failure (%)	6.6 (*n* = 273)	44.5 (n = 1623)	24.3 (*n* = 1896)
Transfusion before ICU (%)	2.9 (*n* = 257)	14.3 (n = 919)	7.7 (*n* = 1176)
Mass transfusion ≥ 10 units (%)	0.1 (*n* = 9)	1.2 (n = 79)	0.6 (*n* = 88)
Thromboembolic event (%)	1.7 (*n* = 69)	7.2 (n = 256)	4.2 (*n* = 325)
Hospital LOS (days), median [IQR]	14 [10–19]	29 [22–42]	18 [11–28]
ICU LOS (days), median [IQR]	6 [3–9]	21 [15–29]	6 [2–16]

*LOS* length of stay, *SD *standard deviation

We identified 12 significant risk factors for a prolonged hospital or ICU stay using logistic regression analysis (Table 5). The need for mechanical ventilation (OR 2.55), and especially the prolonged need for ventilation (OR 37.8), is most important for an prolonged stay. Complications like sepsis (OR 4.12), multi organ failure (OR 1.85), or the occurrence of thromboembolic events (OR 2.72) predicted a longer stay. Extremity fractures also increased the risk for a prolonged stay significantly (OR for legs 2.34; OR for arms 1.37). Injuries to the head, the thorax, or the abdomen were less important, after adjustment.

**Table 5:** Adjusted risk factors for prolonged ICU or hospital LOS, identified with multivariate logistic regression analysis. Risk factors were sorted according to their decreasing importance (odds ratio)

Risk factor	Odds Ratio	95% CI for OR	*p*-value
Mechanical ventilation > 10 days	37.86	30.66—46.69	< .001
Sepsis	4.11	3.04—5.62	< .001
Attempted suicide	2.96	1.86—4.73	< .001
Thromboembolic event	2.72	1.92—3.86	< .001
Mechanical ventilation	2.55	2.22—2.93	< .001
Injury of the legs (AIS 2 +)	2.34	2.00—2.75	< .001
Transfusion required	1.94	1.52—2.48	< .001
Multiple organ failure	1.85	1.53—2.23	< .001
Pelvic injury (AIS 2 +)	1.74	1.47—2.04	< .001
Injury of the arms (AIS 2 +)	1.37	1.20—1.57	< .001
Pre-injury ASA 3 / 4	1.20	1.05—1.36	.006
Male sex	1.18	1.04—1.34	.009
Relevant adominal injury (AIS 2 +)	1.13	0.90—1.43	.293
Relevant head injury (AIS 3 +)	1.08	0.92—1.26	.358
Low fall	1.06	0.93—1.22	.389
Relevant thorax trauma (AIS 3 +)	0.88	0.76—1.02	0.08

*CI* confidence interval, *AIS* abbreviated injury scale, *ASA* American society of anesthesiologists

## Discussion

In the present study, we were able to demonstrate that numerous independent risk factors in the polytraumatized elderly patient exist that contribute to a prolonged inpatient stay. In addition to extended ventilation time, the occurrence of organ failure and thromboembolic events, lower extremity injuries and suicide attempts were identified as risk factors that might possibly be influenced by further improvements in terms of prophylactic and therapeutic interventions, but also organizational and structural adjustments in the care of multiply injured elderly.

An internationally accepted distinct age threshold for the definition of a geriatric trauma patient remains outstanding which makes it difficult to clearly define a geriatric collective of trauma patients. Nevertheless, it is indisputable that elderly patients should be considered as a special group with multiple peculiarities. In this context, numerous studies have demonstrated a relationship between increasing age and mortality following trauma [14]. An in-house mortality of over 60% has been reported for multiply injured patients older than 85 years of age which is significantly higher than for younger patients [15]. Once surviving the initial trauma elderly patients are threatened with ongoing inactivity and long-term changes in their life conditions as well as long hospital stays and extended periods of rehabilitation [16, 17][18]. Based on observable physiological changes and effects on outcome, Advanced Trauma Life Support (ATLS®) recommends treatment in specialized trauma centers from age > 55 years [3]. These expectations can be supported by studies comparing the outcome before and after the implementations of specialized geriatric trauma centers [19]. Multiple studies defined the threshold for a geriatric population by an age of older than 65 years. Regardless of the incongruent age definition in multiple studies, the data presented here clearly meets the criteria of a geriatric collective with a mean age of > 75 years of age.

Length of hospital stay in trauma is identified as one of the main cost factors in the treatment of severely injured patients [20]. In addition, prolonged ICU-LOS is also known to be independently associated with increased mortality and the risk to sustain an adverse event increases by 6% per hospital day [21–23]. For these reasons, we focused on identifying factors that may condition a prolonged stay. Some studies describe disproportionate early patient death as a bias to predict those factors, due to the fact, that patients decease before they even reach the threshold for a prolonged stay. For this reason, we deliberately excluded patients who died prior to our threshold for a prolonged stay from the present study [24]. Once the threshold for prolonged stay was exceeded, mortality in our collective was 15.2%, which is slightly higher than described in age-independent collectives and again emphasizes the higher mortality of older patients [7]. However, no final statement can be made about the functional outcome from the registry data. Apart from mortality, due to the structure of this registry study, we are unable to make an informed statement about the long-term outcome of patients.

Comparable to threshold in regard of the definition of a geriatric population, there is disagreement about the definition for a prolonged inpatient stay or a prolonged intensive care stay, respectively. This complicates the definition and especially the comparability with the few existing studies analyzing prolonged ICU and hospitals stays in trauma. Recent studies use definitions that are arbitrarily set at, for example, 10 days as well as standard lengths of stay that are defined depending on the diagnosis [25]. We based our threshold for a prolonged stay on the average length of stay of the overall collective and defined it belonging to the top 80 percentage. In an age-independent study by Hwarjibe, for example, the threshold was 21 days [25]. Nonetheless, the rate of long-layers in this study was only 5%, it should be noted the ISS was markedly lower than in our patients (ISS mean 18 (12) in the prolonged group). Despite different definitions of prolonged in-hospital stays, this may indicate that prolonged LOS is more common in geriatric patients.

It is generally known that trauma patients make up a relatively small proportion of intensive care patients but consume a high level of resource commitment and have comparatively long intensive care courses [11]. Nevertheless, good outcomes have been described for trauma patients despite longer hospital stays [24]. Previously, high ISS, TBI, co-morbidities, and the occurrence of complications during the inpatient stay were identified as predictors of long hospital stays following trauma [12, 26]. Apart from that, a matched pair study detected that geriatric patients sustain more complications than their younger counterparts [27]. In one of the few studies looking at prolonged hospitalization of trauma patients, Trottier et al. identified higher ISS as a risk factor in addition to advanced age [28]. Our patients with prolonged stays also showed higher ISS, but this was not confirmed as an independent risk factor going along with a comparable mortality during intensive care hospitalization irrespective of the underlying fracture as shown in another study [29]. Mechanical ventilation, particularly prolonged ventilation times for more than 10 days and the development of complications such as sepsis and multiorgan failure were strongly associated with prolonged stay. These findings are supported by a study that has described prolonged ventilation was as a factor for extended ICU-stays in trauma [30]. At the same time, higher age is also known to be a risk factor for prolonged mechanical ventilation [31]. Although higher age was also hypothesized to be a risk, multivariate analysis in the mentioned study showed that age alone could not be considered as sole risk factor for delayed discharge. In this study, the development of urinary tract infections, sepsis and deep vein thrombosis were identified as potentially influenceable risk factors [24]. In a recent study, Spering et al. demonstrated that multiorgan failure–which was a risk factor for prolonged stay in our study—is more common in elderly trauma patients, reaffirming the necessity to take care of this complication in the elderly [1].

In addition to a serious injury of especially the lower extremities, attempted suicide was also identified as a cause for late discharge in our work. In clinical practice, transfer to psychiatric hospitals after trauma as well as transfer to rehabilitation clinics is often complicated when mobility of the patient is still insufficient. The present results confirm these clinical impressions and can thus be identified as a structural problem at the transition between acute and subacute treatment. Interestingly, this observation with problems to organize a subacute stay which then leads to a medically unnecessary elongation of the inpatient stay has been confirmed in an age-independent analysis for trauma patients. Here, 46% of extended stays were attributed to this factor, equaling an odd’s ratio of about 5% [25]. Consequently, there appears to be a fundamental potential for improvement here, as discharge delays to post-acute care facilities have been described as a risk factor for prolonged hospitalization in non-trauma patients as well [32] [33, 34].

We demonstrated that the need for RBC administration during the resuscitation in the emergency department was associated with an increased risk of prolonged stay. This is consistent with the results of several studies in surgical patients in which an association between the administration of blood products and prolonged stay was also found [35]. However, the present results must be interpreted with caution; a definite statement as to whether the administration of blood products is causative or associative with a prolonged stay cannot be made with certainty from the data available here.

## Limitations

Certain limitations of this investigation must be acknowledged. As the present study is a retrospective registry analysis, all findings represent associations and do not claim any causality. There may be some predictors for length of stay, which we cannot correct for since they were not documented in the registry. Although the data quality in the TR-DGU is high based on multiple checks on data entry, registry data are general less valid than data from prospective randomized trials. Long-term data beyond discharge were not available, and the structure of the registry did not allow to perform follow-up examinations. Additionally, the mortality in the prolonged stay group may be conditioned in part by the existence of and compliance with an advance directive [36]. To make results more comparable, an overall definition for prolonged stay would be necessary.

## Conclusion

In the present study, independent risk factors associated with prolonged hospitalization were identified for severely injured elderly patients. In addition to mechanistic criteria (e.g. suicide attempt) and specific injury patterns such as severe lower extremity injuries, logistic influences such as the interface with further rehabilitation could be identified as risk factors. Therefore, in addition to attention to an improvement in clinical treatment, structural changes in patient care should be included in further planning, as well.

## Data Availability

Availability of data and materials If necessary, the corresponding data sets on which the analyses are based can be requested from the corresponding author.

## Abbreviations

AIS: Abbreviated injury scale
ASA: American society of anesthesiologists
ATLS: Advanced trauma life support
AUC: Academy for trauma surgery
CI: Confidence interval
DOAC: Direct acting oral anticoagulants
DGU: German society of trauma surgery
GAS: Germany, Austria, Switzerland
GCS: Glasgow coma scale
ICU: Intensive care units
IMC: Intermediate care
INR: International normalized ratio
ISS: Injury severity score
LOS: Length of stay
NIS: Committee on emergency medicine, intensive care and trauma management
OR: Odds ratio
PAI: Platelet aggregation inhibition
PTT: Partial thromboplastin time
RISC: Revised injury severity classification
SBP: Systolic blood pressure
SD: Standard deviation
TBI: Traumatic brain injury
TR-DGU: Trauma registry of the German trauma society

## References

[1] Spering C, Lefering R, Bouillon B et al (2020) It is time for a change in the management of elderly severely injured patients! An analysis of 126,015 patients from the traumaregister DGU®. Eur J Trauma Emerg Surg 46:487–497 31520156 10.1007/s00068-019-01229-8

[2] Madni TD, Ekeh AP, Brakenridge SC et al (2017) A comparison of prognosis calculators for geriatric trauma. J Trauma Acute Care Surg 83:90–96 28422904 10.1097/TA.0000000000001506

[3] Bonne S, Schuerer DJE (2013) Trauma in the older adult: epidemiology and evolving geriatric trauma principles. Clin Geriatr Med 29:137–150 23177604 10.1016/j.cger.2012.10.008

[4] Lin P-C, Wu N-C, Su H-C et al (2022) Comprehensive comparison between geriatric and nongeriatric patients with trauma. Medicine 101:e28913 35363212 10.1097/MD.0000000000028913 PMC9281953

[5] Kalbas Y, Lempert M, Ziegenhain F et al (2021) A retrospective cohort study of 27,049 polytraumatized patients age 60 and above: identifying changes over 16 years. Eur Geriatr Med 13:233–241 34324144 10.1007/s41999-021-00546-9 PMC8860799

[6] Bardes JM, Benjamin E, Schellenberg M et al (2019) Old age with a traumatic mechanism of injury should be a trauma Team activation criterion. J Emerg Med 57:151–155 31078345 10.1016/j.jemermed.2019.04.003

[7] Kisat MT, Latif A, Zogg CK et al (2016) Survival outcomes after prolonged intensive care unit length of stay among trauma patients: the evidence for never giving up. Surgery 160:771–780 27267552 10.1016/j.surg.2016.04.024

[8] Hashmi A, Ibrahim-Zada I, Rhee P et al (2014) Predictors of mortality in geriatric trauma patients: a systematic review and meta-analysis. J Trauma Acute Care Surg 76:894–901 24553567 10.1097/TA.0b013e3182ab0763

[9] Mörs K, Wagner N, Sturm R et al (2021) Enhanced pro-inflammatory response and higher mortality rates in geriatric trauma patients. Eur J Trauma Emerg Surg 47:1065–1072 31875239 10.1007/s00068-019-01284-1

[10] Lefering R, Huber-Wagner S, Nienaber U et al (2014) Update of the trauma risk adjustment model of the TraumaRegister DGU™: the revised injury severity classification, version II. Crit Care 18:476 25394596 10.1186/s13054-014-0476-2 PMC4177428

[11] Laupland KB, Kirkpatrick AW, Kortbeek JB et al (2006) Long-term mortality outcome associated with prolonged admission to the ICU. Chest 129:954–959 16608944 10.1378/chest.129.4.954

[12] Kashkooe A, Yadollahi M, Pazhuheian F (2020) What factors affect length of hospital stay among trauma patients? A single-center study. Southwestern Iran Chin J Traumatol 23:176–180 32171653 10.1016/j.cjtee.2020.01.002 PMC7296356

[13] Sánchez Arteaga A, Tinoco González J, Tallón Aguilar L et al (2022) Long-term influence of frailty in elderly patients after surgical emergencies. Eur J Trauma Emerg Surg 48:3855–3862 34741180 10.1007/s00068-021-01818-6

[14] van Wessem KJP, Leenen LPH (2022) Geriatric polytrauma patients should not be excluded from aggressive injury treatment based on age alone. Eur J Trauma Emerg Surg 48:357–365 33320284 10.1007/s00068-020-01567-y PMC7736672

[15] de Vries R, Reininga IHF, de Graaf MW et al (2019) Older polytrauma: mortality and complications. Injury 50:1440–1447 31285055 10.1016/j.injury.2019.06.024

[16] Siracuse JJ, Odell DD, Gondek SP, Odom SR, Kasper EM, Hauser CJ, et al. Health care and socioeconomic impact of falls in the elderly. Am J Surg. 2012;203:335–8; discussion 338. 10.1016/j.amjsurg.2011.09.018 22257741

[17] Miyoshi Y, Kondo Y, Hirano Y et al (2020) Characteristics, injuries, and clinical outcomes of geriatric trauma patients in Japan: an analysis of the nationwide trauma registry database. Sci Rep 10:19148 33154440 10.1038/s41598-020-76149-4 PMC7645585

[18] de Vries R, Reininga I, de Graaf M, Banierink H, Bosma E, Munzebrock A, et al. 2022 The effect of age on resilience of health-related quality of life among polytrauma patients: a cross-sectional multicenter study. Eur J Trauma Emerg Surg. 10.1007/s00068-022-02135-2 PMC10175333 36416946

[19] Halvachizadeh S, Gröbli L, Berk T et al (2021) The effect of geriatric comanagement (GC) in geriatric trauma patients treated in a level 1 trauma setting: a comparison of data before and after the implementation of a certified geriatric trauma center. PLoS ONE 16:e0244554 33428650 10.1371/journal.pone.0244554 PMC7799827

[20] van der Vlegel M, Haagsma JA, Havermans RJM et al (2021) Long-term medical and productivity costs of severe trauma: Results from a prospective cohort study. PLoS ONE 16:e0252673 34086788 10.1371/journal.pone.0252673 PMC8177462

[21] Moitra VK, Guerra C, Linde-Zwirble WT et al (2016) Relationship between ICU length of stay and long-term mortality for elderly ICU survivors. Crit Care Med 44:655–662 26571190 10.1097/CCM.0000000000001480 PMC4792682

[22] Chaudhary MA, Schoenfeld AJ, Koehlmoos TP et al (2019) Prolonged ICU stay and its association with 1-year trauma mortality: an analysis of 19,000 American patients. Am J Surg 218:21–26 30722934 10.1016/j.amjsurg.2019.01.025

[23] Andrews LB, Stocking C, Krizek T et al (1997) An alternative strategy for studying adverse events in medical care. Lancet 349:309–313 9024373 10.1016/S0140-6736(96)08268-2

[24] Ong AW, Omert LA, Vido D et al (2009) Characteristics and outcomes of trauma patients with ICU lengths of stay 30 days and greater: a seven-year retrospective study. Crit Care 13:R154 19778422 10.1186/cc8054 PMC2784377

[25] Hwabejire JO, Kaafarani HMA, Imam AM, Solis C v., Verge J, Sullivan NM, et al. 2013 Excessively Long Hospital Stays After Trauma Are Not Related to the Severity of Illness. JAMA Surg.148:956. 10.1001/jamasurg.2013.2148 23965602

[26] Moore L, Stelfox HT, Turgeon AF et al (2014) Hospital length of stay after admission for traumatic injury in Canada: a multicenter cohort study. Ann Surg 260:179–187 24646540 10.1097/SLA.0000000000000624

[27] Jensen KO, Lempert M, Sprengel K et al (2020) Is there any difference in the outcome of geriatric and non-geriatric severely injured patients?—A seven-year, retrospective, observational cohort study with matched-pair analysis. J Clin Med 9:3544 33153102 10.3390/jcm9113544 PMC7692238

[28] Trottier V, McKenney MG, Beninati M et al (2007) Survival after prolonged length of stay in a trauma intensive care unit. J Trauma 62:147–150 17215746 10.1097/01.ta.0000250496.99127.4a

[29] Knauf T, Jensen KO, Hack J et al (2020) Type of underlying fracture after the surgical treatment of geriatric trauma patients has no effect on mortality during intensive care treatment. Geriatr Gerontol Int 20:1120–1125 33155420 10.1111/ggi.14053

[30] Kung S-C, Lin W-T, Tsai T-C et al (2017) Epidemiologic characteristics and outcomes of major trauma patients requiring prolonged mechanical ventilation. Medicine 96:e9487 29384944 10.1097/MD.0000000000009487 PMC6393113

[31] Agle SC, Kao LS, Moore FA et al (2006) Early predictors of prolonged mechanical ventilation in major torso trauma patients who require resuscitation. Am J Surg 192:822–827 17161101 10.1016/j.amjsurg.2006.08.051

[32] Doctoroff L, Herzig SJ (2020) Predicting patients at risk for prolonged Hospital Stays. Med Care 58:778–784 32826743 10.1097/MLR.0000000000001345 PMC7444462

[33] Zhao EJ, Yeluru A, Manjunath L et al (2018) A long wait: barriers to discharge for long length of stay patients. Postgrad Med J 94:546–550 30301835 10.1136/postgradmedj-2018-135815

[34] Wilson DM, Vihos J, Hewitt JA et al (2013) Examining waiting placement in hospital: utilization and the lived experience. Glob J Health Sci 6:12–22 24576361 10.5539/gjhs.v6n2p12 PMC4825473

[35] Veenith T, Sharples L, Gerrard C et al (2010) Survival and length of stay following blood transfusion in octogenarians following cardiac surgery. Anaesthesia 65:331–336 20148816 10.1111/j.1365-2044.2009.06225.x

[36] Schindler CR, Woschek M, Verboket RD et al (2020) Registry-based mortality analysis reveals a high proportion of patient decrees and presumed limitation of therapy in severe geriatric trauma. J Clin Med. 10.3390/jcm9092686 32825084 10.3390/jcm9092686 PMC7565431

